# Metaplastic Breast Carcinoma in U.S. Population: Racial Disparities, Survival Benefit of Adjuvant Chemoradiation and Future Personalized Treatment with Genomic Landscape

**DOI:** 10.3390/cancers15112954

**Published:** 2023-05-28

**Authors:** Asad Ullah, Jaffar Khan, Abdul Qahar Khan Yasinzai, Katharine Tracy, Tena Nguyen, Bisma Tareen, Andrea Agualimpia Garcia, Saleh Heneidi, Sheila E. Segura

**Affiliations:** 1 Department of Pathology, Immunology, and Microbiology, Vanderbilt University, Nashville, TN 37232, USA; 2 Department of Pathology, Indiana University School of Medicine, Indianapolis, IN 46202, USA; 3 Department of Medicine, Bolan Medical College, Quetta 83700, Pakistan; 4 Medical College of Georgia, Augusta University, Augusta, GA 30912, USA; 5 Department of Pathology & Immunology, Baylor College of Medicine, Houston, TX 77030, USA; 6 Department of Pathology, Cedars Sinai Medical Center, Los Angeles, CA 90048, USA

**Keywords:** metaplastic breast carcinoma, SEER database, chemotherapy

## Abstract

**Simple Summary:**

Metaplastic breast carcinoma (MBC) is a rare, heterogenous group of aggressive triple-negative breast cancers with a characteristically poor prognosis and response to standard treatments. The rarity of MBC greatly limits insight into the clinical presentation, management, and scientific investigation. We aimed to evaluate the demographics and characteristics of MBC as well as survival outcomes based on presentation and treatment. Several features regarding tumor size, grade, stage, and patient age were found to be correlated with worse prognosis while the best overall survival was seen in patients who underwent combined surgery, chemotherapy, and radiotherapy. Black patients in the study had significantly worse outcomes than White patients, with higher rates of aggressive tumor features at presentation. The most common genetic mutations observed were TP53, PIK3CA, and LRP1B.

**Abstract:**

Purpose: In this population-based study, we aim to identify factors that are influential on the survival outcome in MBC and investigate novel molecular approaches in personalized disease management. Methods: The data of this study were collected from the SEER database from 2000–2018. A total of 5315 cases were extracted from the database. The data were evaluated for demographics, tumor characteristics, metastasis, and treatment. Survival analysis was completed by using SAS software for multivariate analysis, univariate analysis, and non-parametric survival analysis. The molecular data with the most common mutations in MBC were extracted from the Catalogue of Somatic Mutations in Cancer (COSMIC) database. Results: The mean age at the time of presentation was 63.1 with a standard deviation (SD) of 14.2 years. Most patients were White (77.3%) with 15.7% Black patients, 6.1% Asian or Pacific Islander, and 0.5% American Indian. Histologically, most of the reported tumors were grade III (74.4%); 37% of the cases were triple negative (ER-, PR- and HER2-), whereas the hormone status was unknown in 46% of the cases. Spread was localized in 67.3% of patients while 26.3% had regional spread and 6.3% had distant metastases. Most tumors were unilateral (99.9%) and between 20–50 mm in size (50.6%). The lungs were the most common site for distant metastasis at diagnosis (3.42%) followed by bone (1.94%), liver (0.98%), and brain (0.56%). A combination of surgery, chemotherapy, and radiation therapy was the most common treatment with a cause-specific survival rate of 78.1% (95% CI = 75.4–80.4). The overall survival rate at 5 years was 63.6% (95% confidence interval (CI) = 62.0–65.1) with a cause-specific survival of 71.1% (95% CI = 69.5–72.6). Cause-specific survival was found to be 63.2% (95% CI = 58.9–67.1) in Black patients as compared to 72.4% (95% CI = 70.1–74.1) in White patients. Black patients also presented with higher rates of grade III disease, distant metastasis, and larger tumor size. On multivariate analysis, age > 60, grade III+, metastasis, and tumor size > 50 mm were associated with worse survival. The most common mutations in MBC identified in COSMIC data were TP53, PIK3CA, LRP1B, PTEN, and KMT2C. Conclusion: Though rare, MBC is aggressive, with poor prognosis associated with high-grade tumors, metastasis, tumor size over 50 mm, and advanced age at the time of presentation. Overall, Black women had worse clinical outcomes. MBC is difficult to treat and carries a poor prognosis that affects various races disproportionately. Continued enhancement of treatment strategies to foster more individualized care as well as continued enrollment in clinical trials are needed to improve outcomes among patients with MBC.

## 1. Introduction

Metaplastic breast carcinoma (MBC) is invasive breast cancer that accounts for less than 10,000 cases annually, making up only 0.2–5% of breast cancers diagnosed worldwide [1]. It most commonly presents clinically as a quickly growing unilateral breast mass with either irregular or circumscribed appearance on imaging and usually without the presence of calcifications [2,3]. The literature reports that 70–90% of MBC cases are triple-negative, in that they do not express receptors for estrogen (ER), progesterone (PR), and human epidermal growth factor 2 (HER2) [4,5,6]. MBC confers the worst prognosis compared to other types of breast cancers, often presenting with large tumors and locally advanced disease at the time of diagnosis [7,8]. Aside from the aggressive nature of MBC, the rarity of these tumors in addition to the heterogeneous histological findings make the diagnosis more challenging, contributing to the poor prognosis of the disease [9].

As most breast cancers are composed of glandular epithelium, breast metaplasia is defined as the transition to a non-glandular cell type physiologically absent in healthy breast tissue [10]. Therefore, MBC is characterized as a heterogeneous group of histological subtypes composed of epithelial and mesenchymal cellular morphologies [4]. Three broad categories of MBC include epithelial-only, biphasic, and monophasic (pure) carcinomas [11]. The epithelial-only category includes low-grade adenosquamous and squamous cell carcinomas [12]. Mixed metaplastic carcinoma encompasses the biphasic category in that it contains both epithelial and sarcomatoid components [12]. The monophasic (pure) category includes spindle cell carcinoma, fibromatosis-type spindle cell carcinoma, and metaplastic carcinoma with mesenchymal differentiation [12]. The rarity of MBC and the subsequently limited research poses a challenge in determining the best treatment for MBC and its subtypes. Currently, MBC is treated following the guidelines for triple-negative breast carcinoma (TNBC); however, this approach has shown minimal efficacy [13,14]. Further understanding of the unique pathogenesis and clinical presentation of MBC is needed to improve treatment outcomes for this disease. In this study, we utilized the SEER database to analyze demographic, treatment, and racially stratified outcomes and investigated potential avenues for therapeutic advancement in MBC.

## 2. Materials and Methods

Initiated in 1972by the National Cancer Institute, the Surveillance, Epidemiology and End Results (SEER) database now covers approximately 28% of the US population. The SEER*Stat software (Version 8.4.0) was used to collect data from 2000–2018 using the International Classification of Diseases version 3 (ICD-O-3) (https://seer.cancer.gov/seerstat/, accessed on 15 January 2023). There were 18 registries in the SEER database selected for this analysis. The SEER-18 registry include geographic regions: Alaska, Atlanta, California, Connecticut, Detroit, Georgia, Hawaii, Iowa, Kentucky, Louisiana, New Mexico, New Jersey, Seattle, and Utah (https://seer.cancer.gov/data-software/documentation/seerstat/nov2020/, accessed on 15 January 2023). Kaplan–Meier graphs were generated by the Statistical Analysis System (SAS).

Extracted data included age, race, tumor grade, tumor size, tumor stage, lymph node status, metastasis, treatment modality, and overall survival stratified by these metrics. Cases of MBC were confirmed microscopically with positive histology, immunophenotyping, genetic studies, and/or confirmed microscopically with an unspecified method. Data were collected across several settings including inpatient facilities, clinics, laboratories, private practices, nursing homes, and hospice. Cases excluded from our study were those with positive laboratory test/marker studies, direct visualization without microscopic confirmation, radiography without microscopic confirmation, clinical diagnosis only, data only from autopsy or death certificate, and cases with unknown status. The cases selected for survival analysis were those with microscopic confirmation, malignant behavior, and patients with a known age. 

This study used the chi-square test to check for significant associations, non-parametric and parametric cox regression methods for survival analysis to produce Kaplan graphs and calculate hazard ratios (HR), and to identify the independent factors that affect survival. Data that were either unidentified or missing were removed from multivariate analysis. Univariate cox regression analysis was used to screen significant factors for a multivariate model with an accepted *p*-value of <0.25. This was done to reduce the risk of adding irrelevant and non-significant variables into the final multivariate cox regression model, which can lead to overfitting and unreliable results. Multivariate cox regression analysis was then used to analyze the data, and statistical significance was defined as *p* < 0.05.

In total, 5315 cases of metaplastic carcinoma of the breast of were identified from 2000–2018. The molecular data with common mutations in MBC were extracted from the Catalogue of Somatic Mutations in Cancer (COSMIC) database.

### 2.1. Demographic Data and Tumor Characteristics

**Figure 1:**
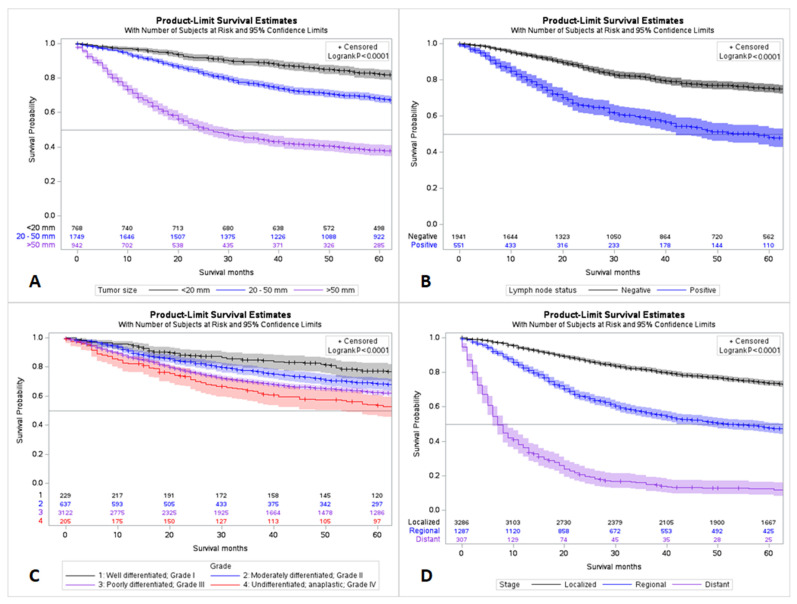
Survival trends stratified by tumor size (**A**), grade (**B**), lymph node status (**C**), and stage at diagnosis (**D**).

The mean age at the time of diagnosis was found to be 63.1 years, with a standard deviation (SD) of ±14.2 years. Most of the patients identified as White (n = 4106, 77.3%) for race, followed by Black (n = 833, 15.7%), Asian or Pacific Islander (n = 324, 6.1%), and American Indian or Alaska Native (n = 27, 0.5%). Of the known cases, 74.4% were poorly differentiated (grade—III) and 4.9% were undifferentiated/anaplastic (grade—IV). The receptor status included the estrogen receptor (ER), progesterone receptor (PR), which are together represented by hormone receptors (HR), and the human epidermal growth factor receptor 2 (HER2). The data for HER2 were available from 2010 onwards. When the hormone status was known, the data shows that the largest group of cases (37.2%) were triple negative. The remaining cases were classified into one of three categories: 13.1% expressed HR (both ER and PR) but not HER2, 2% expressed HER2 but not HR, and 1.1% expressed both HR and HER2. About (46.5%) had borderline or unknown receptor status. Most cases were localized (67.3%) with regional spread in 26.3% of cases and distant spread in 6.3%. The tumor size was unknown in 1856 (34.9%) cases and known in 3459 (65.0%) cases. Most tumors were between 20–50 mm (50.6%) in size. Patients with large tumor size, node-positive disease, undifferentiated/grade IV disease, and distant metastasis were associated with a worse clinical outcome (*p* < 0.001) (Table 1 and Figure 1). 

### 2.2. Distant Metastasis and Lymph Node Status at the Time of Diagnosis of Metaplastic Carcinoma of the Breast

**Figure 2:**
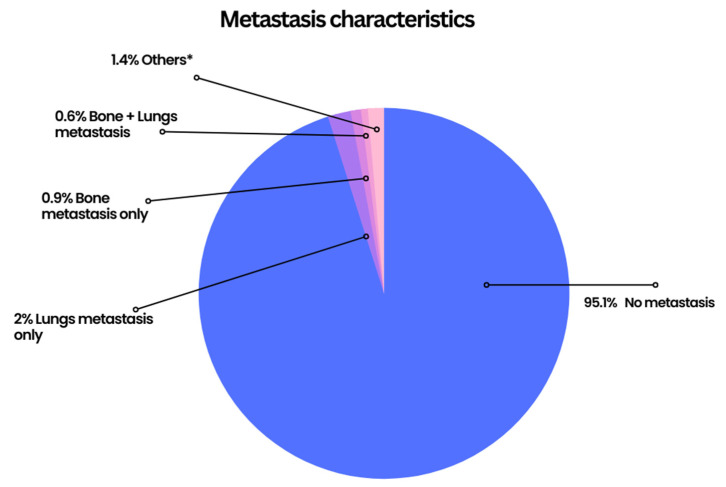
Pie chart demonstrating the patients’ metastatic status of MBC. * Rare combinations of metastasis or mostly statuses were unknown.

Positive lymph nodes were reported in 22.11% of the known cases. Of the 3040 patients with known metastasis status, 95.1% had no metastasis, 3.6% had metastasis to a single site (bone, brain, liver, or lung), and 1.6% had metastasis involving multiple sites. The lungs were found to be the most common site of metastasis with a frequency of 3.42% with 2% presenting as a solitary lung lesion (Figure 2 and Supplementary Table S1). 

### 2.3. Treatment Characteristics

**Figure 3:**
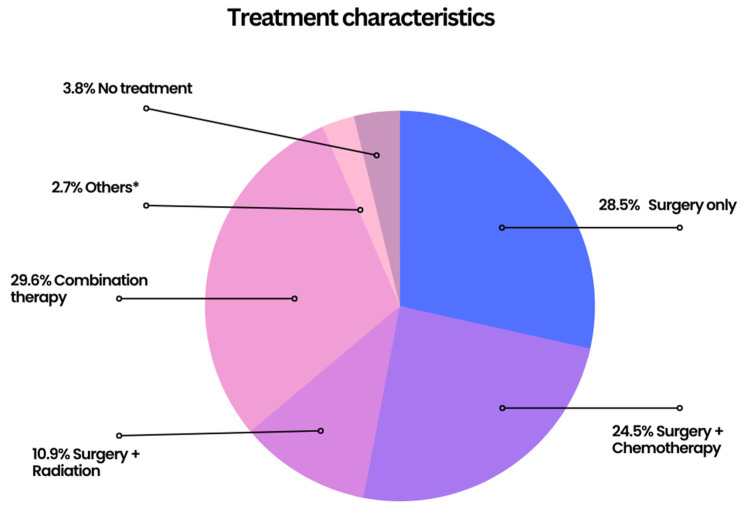
Pie chart for different treatment modalities of MBC. * rare combinations of treatments or statuses for multiple modalities are unknown.

Only 199 (3.8%) patients did not undergo any treatment. Surgery only was used in 1509 (28.5%) cases, while 125 (2.4%) cases used chemotherapy alone, and a mere 6 (0.1%) used radiation alone. Combination therapy with surgery and chemotherapy was used in 1296 (24.5%) cases, while 574 (10.9%) patients had surgery plus radiation, and just 11 (0.2%) patients had chemotherapy plus radiation without surgery as a treatment. Combination treatment with surgery, chemotherapy, and radiation was utilized in 1568 (29.6%) cases. Treatment status was unknown in just 24 (0.5%) cases and was known in 5291 (99.5%) cases (Figure 3 and Supplementary Table S2).

### 2.4. Outcomes and Survival Analysis

**Figure 4:**
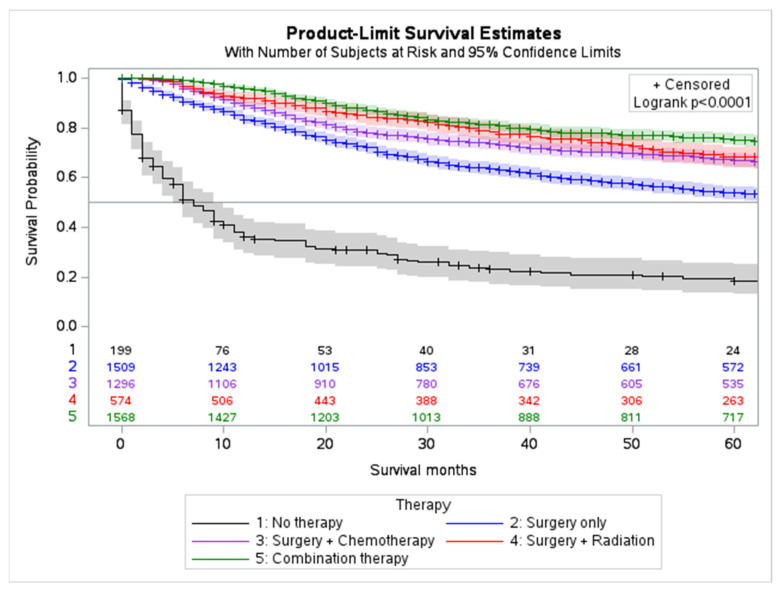
Overall survival trends stratified by treatment regimens.

The overall 5-year survival rate was 63.6% (C.I. 95%, 62.0–65.1, while the cause-specific 5-year survival rate was 71.1% (C.I. 95%, 69.5–72.6). Based on treatment modality, the 5-year survival rate for those treated with chemotherapy, surgery, and radiation only, was 72.6% (C.I. 95%, 70.6–74.5), 73.6% (C.I. 95%, 72.0–75.1), and 78.7% (C.I. 95%, 76.5–80.7), respectively. For those treated with combined surgery, chemotherapy, and radiation, the 5-year survival rate was 78.1% (C.I. 95%, 75.4–80.4). Combination therapy (surgery and adjuvant chemotherapy) are associated with the best overall outcome followed by surgery and adjuvant radiation (*p* < 0.001) (Figure 4 and Supplementary Table S3).

### 2.5. Survival Analysis by Race

**Figure 5:**
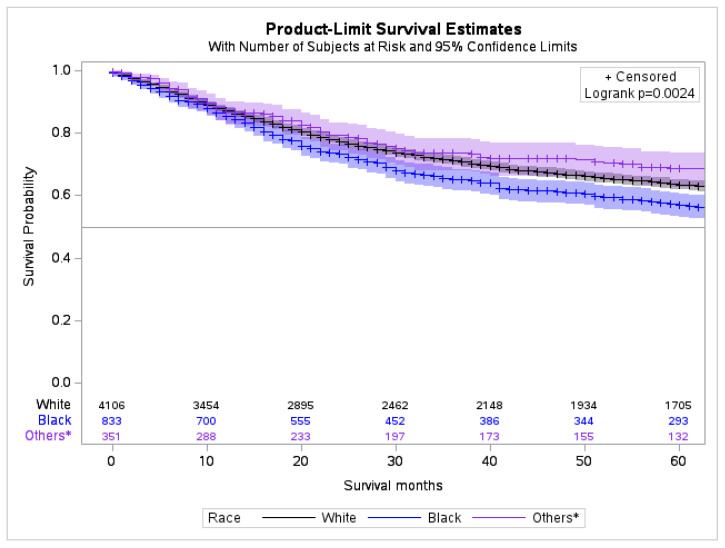
Overall survival trends of MBC stratified by race. * Asian, Pacific Islander, Indian American, and Alaska Native.

The cumulative 5-year survival rate for patients of White, Black, Asian/Pacific Islander, and American Indian/Alaska Native race was 72.4% (C.I. 95%, 70.7–74.1), 63.2% (C.I. 95%, 58.9–67.1), 75.2% (C.I. 95%, 68.7–80.6), and 63.3% (C.I 95%, 37.9–80.6), respectively. The Black race had the lowest survival outcome compared to White Americans and Native/Alaskans (*p* < 0.0024) (Figure 5, Supplementary Table S4). There was no significant difference noted for the race and hormone receptors statuses. The different variables for racial disparities for the largest cohort of the US population, i.e., White and Black Americans, are compared in Table 2. 

### 2.6. Survival Difference by Stage for Race and Treatment

**Figure 6:**
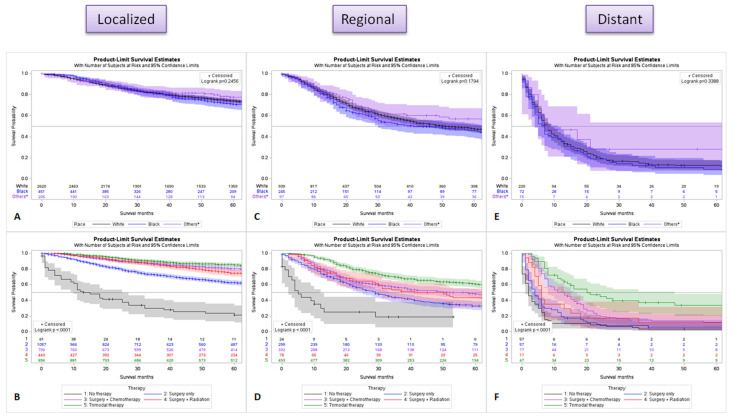
Survival analysis for race and treatment modalities for localized (**A**,**B**), regional spread (**C**,**D**) and distant metastatic disease (**E**,**F**). * Asian and American Indians, Pacific Islander, and Alaska Native.

In localized (organ-confined) disease, the Black race is associated with slightly lower survival in comparison to other races (Figure 6A), and the highest survival was observed when patients received all three modalities of treatment (surgery, chemotherapy, and radiation) (Figure 6B). In regional spread (direct extension to nearby or regional lymph node spread) disease, Asian and American Indians, and Alaska Native (others) had the best overall long-term survival compared to the White and Black races (Figure 6C), and trimodality therapy had the best clinical outcome (Figure 6D). In distant disease (distant metastatic disease), there was no clinically significant survival difference for race (Figure 6E), and multimodal therapy with surgery, chemotherapy, and radiation remains the best treatment (Figure 6F). Overall, surgery with chemotherapy and radiation is associated with the best survival in all stages of the disease. 

### 2.7. Multivariable Analysis

Cox survival regression was utilized to perform multivariable analysis for the impact of various factors on mortality. The analysis identified age > 60 years hazard ratio (HR) (HR 1.958, *p* = 0.001), undifferentiated/anaplastic—grade IV (HR 3.692, *p* = 0.002), distant spread (HR 2.613, *p* = 0.012), size > 50 mm (HR 3.275, *p* = 0.001), and brain metastasis (HR 29.266, *p* = 0.001) as factors associated with increased mortality (Table 3).

## 3. Mutations Associated with Metaplastic Breast Carcinoma in the COSMIC Database

The genetic mutations associated with metaplastic breast carcinoma (MBC) were extracted from COSMIC (https://cancer.sanger.ac.uk/cosmic, accessed on 25 April 2023) version GRCh37 COSMIC v97. A total of 16,560 cases of breast carcinoma were evaluated for genetic mutations in the database. In the sub-selection category, all the cases including nipple (15), extramammary (91), and not otherwise specified (NOS) (16,454) cases in both breasts were selected. A total of 14,964 cases of carcinoma were selected and 285 cases of metaplastic breast carcinoma were identified in the database. The data for the top 20 mutations in order of frequency were TP53 59% (in total 281 samples tested), PIK3CA 34% (289), LRP1B 11% (106), PTEN 10% (287), KMT2C 9% (119), PIK3R1 7% (150), KMT2D 7% (145), RB1 6% (200), NF1 6% (141), PTPRT 6% (117), APC 5% (265), KDM6A 5% (153), ARID1A 5% (140), MAP3K1 5% (119), FAT1 5% (117), HRAS 3% (253), CTNNB1 3% (247), FBXW7 2% (266), JAK3 2% (266), and RET 2% (216).

## 4. Discussion

The results of this study represent the largest data set of MBC cases in the United States and is likely the most accurate reflection of the true epidemiology of the disease. We found a 5-year overall survival rate of 63.60% (95% CI = 62.0–65.1) across all study demographics but found significant differences among racial groups. Black patients with MBC had a worse survival rate at 5 years (63.20%) compared to White (72.4%) and Asian or Pacific Islander (75.2%) patients. The American Indian or Alaska Native group also had worse survival rates (63.3%), though this may be attributed to a very small sample size compared to the other races. Multivariate analysis of this data indicates that advanced age, grade III+, distant metastasis, and tumor size > 50 mm at presentation confers a worse prognosis. Black patients were found to have increased rates of all these prognostic factors compared to White patients; Black patients were also found to have increased rates of bone, liver, and lung metastases, which all confer a decreased survival. Specifically, rates of brain metastasis were statistically similar in both Black and White patients, though this may be attributed to the small sample size. These findings are consistent with previous studies on non-metaplastic breast cancer in which Black patients have been found to present with tumor features that carry a worse outcome [15,16]. Of these prognostic factors, stage at diagnosis has been found to account for the largest portion of discrepancy in survival rates among racial and ethnic groups [17]. One study investigating potential causes of these racial differences identified insurance status and area-level education attainment as factors impacting prognostic findings [18]. The DeSantis team found adjusting for insurance status and area-level education attainment accounted for 39% of the increased risk for metastasis, 31% of increased tumor size, and 25% of positive lymph nodes in Black patients with breast cancer. Only a 14% reduction was seen in tumor grade when controlling for these socioeconomic factors [18]. Correlating with these findings, a SEER database analysis performed by the same team found Black patients were more often uninsured and living in areas of low educational attainment than White patients [18]. An analysis of the National Cancer Database (NCDB) showed that being uninsured and living in an area with low educational attainment confers worse prognostic factors for breast cancer even when controlling for race and age [18]. A different team found socioeconomic factors explained half of the increased mortality in Black patients compared to other groups [16]. One important consideration is the impact of these factors on breast cancer screening. The utilization of mammograms for breast cancer preventative screening declines with lower household income and decreased educational attainment [19]. Although studies report decreases in mortality of 15–30% due to early detection with mammograms, there is a significant problem with overdiagnosis, leading to debate regarding the true efficacy of this screening method [20]. Most breast cancer is detected by self-examination—a skill that may be negatively influenced by lack of access to preventative breast cancer education, specifically with uninsured patients and individuals with lower levels of formal education [21]. 

A study by Schroeder et al., demonstrated that HER2 positive MBC is associated with a higher survival rate than HER2 negative disease [22]. We found the rate of HER2 positive status to be similar among Black and White patients with no effect on overall survival. Age also plays an interesting role as MBC has a higher incidence in older patients (63 years vs. 61 years in ductal type) with an incidence of 16% in Black women [22]. Several studies have found that Black patients with breast cancer are more likely to present at a younger age than White patients [15]. Our results reflect these findings in MBC with an average age at diagnosis of 64.03 in White patients compared to an average age of 60.12 in Black patients. Studies have found that a younger age at diagnosis in breast cancer usually confers a worse prognosis, though this correlation is not apparent from our SEER data in MBC [23]. Interestingly, our data found that patient age over 60 confers a higher risk of mortality in MBC (HR 1.958) compared to patients younger than 60. This finding may be attributed to the age of 60 being used as a reference for our data, while other studies used 40 as a reference point [23]. However, since the overall average age at presentation is 63.14, this reference point reflects that above-average age may suggest a worse prognosis. 

Metastatic MBC is a strong indicator of poor prognosis with 5-year survival rates ranging from 25% in lymph node-positive disease to 0% for brain and liver metastases. We observed that 4.9% of patients presented with at least one distant metastasis, and 22.1% of cases presented with positive lymph nodes. Our observed frequency of lymph node involvement is congruent with previous studies that found the rate to be between 6% and 28% [24]. The most common site of distant metastases were the lungs in 3.42% of cases, followed by bone, liver, and brain. Although the prognosis of metastatic disease is worse, the incidence of metastasis at the time of diagnosis for MBC is lower than non-metaplastic breast cancer [25]. One study found that within a 46-patient cohort, of which 4.6% of patients presented with metastatic disease, 32.6% went on to develop distant metastases [14]. The propensity of MBC to metastasize after diagnosis combined with the poor outcomes associated with metastatic disease contributes to the poor overall survival of MBC. 

Surgical resection is the mainstay of MBC treatment due to the disease’s locally aggressive nature. Patients with MBC are more likely to receive mastectomy than patients with non-metaplastic breast cancer; however, breast-conserving surgery with post operative radiation may be used if margins allow [6,8,26]. In our study, surgery with adjuvant radiotherapy significantly increased 5-year survival compared to surgery alone (73.6%) with a survival rate close to that of a combined regimen with chemotherapy (79.0% vs. 78.1%). A recent study found that MBC patients who received post-mastectomy radiation had a better breast-cancer-specific survival compared to patients who did not receive radiotherapy [27]. Compared to non-metaplastic breast cancer, chemotherapy has shown limited efficacy but is still used for the treatment of MBC due to some evidence of increased survival [26]. Specifically, some studies indicate that platinum chemotherapy agents may be efficacious against the squamous epithelial subtype of MBC, while doxorubicin or ifosfamide may be efficacious for sarcomatoid MBC [28]. Additionally, *BRCA* mutations may increase sensitivity to platinum agents and should be considered for adjuvant therapy [29]. Our data support that surgery with adjuvant radiation and chemotherapy provides the best outlook for survival in MBC patients; however, information on MBC subtype and *BRCA* mutations may help guide patient management and therapy. 

### Immunotherapy and Molecular Targets

The negative hormone receptor status of MBC narrows current pharmaceutical treatment to chemotherapy. The discovery of new molecular targets is paramount to the advancement of treatment for MBC and is the most promising means of improving survival. Like the heterogeneous histological presentation, MBC has many identifiable genetic aberrations. Studies have found mutations in *TP53* in 65% of MBC patients and *PIK3CA* mutations in 35% [28]. *TP53* mutations have been associated with increased expression of VEGF-A, a molecular target [27]. High rates of mutations and increased phosphorylation of proteins in the *PI3K/AKT/mTOR* pathway have been shown in MBC cases as compared to other TNBC [30,31]. Molecular targets in this pathway are being investigated for efficacy in the treatment of MBC. Studies investigating the impact of a combined regimen of Doxorubicin, Bevacizumab (VEGF inhibitor), and Temsirolimus (mTOR inhibitor) have found some response to therapy, indicating a need for further investigation into utilization of this pathway as a therapeutic target for MBC and TNBC [32,33]. 

The role of macrophages is also being investigated as a possible therapeutic intervention. Tumor associated macrophages (TAMs) are associated with breast cancer cell proliferation, invasion, distant metastasis, and treatment resistance through several mechanisms [34]. It has been reported that high TAM recruitment is associated with worse clinical outcome in patients with breast cancer [34]. Montemurro, et al., demonstrated the role of tumor microenvironment in recurrent glioblastoma, indicating that glioma-associated macrophages (GAMs) are associated with tumor progression and resistance to chemotherapy and radiation [35]. Strategies aimed at inhibiting macrophage recruitment and phagocytosis of macrophage mediated tumor cells will improve survival in breast cancer.

MBC has also been found to exhibit epithelial-to-mesenchymal transition (EMT) and stem-cell-like qualities. These factors contribute to the aggressive nature, poor prognosis, and resistance to chemotherapy of MBC [31,36,37]. A high CD44/CD24 ratio has been implicated in tumorigenic activity seen in MBC, categorizing cells as stem-cell-like cells with advanced differentiation capabilities [31,38]. EMT is characterized by cadherin switching early in its pathogenesis [39]. Studies investigating EMT in MBC found that cadherins normally found in epithelial tissues (E-cadherin) are downregulated, while non-epithelial cadherins (N-cadherin and cadherin-11) are overexpressed [38,39]. Transcription factors that induce EMT, including SNAIL, ZEB, and TWIST, work together to decrease E-cadherin expression while upregulating N-cadherin in an intricate network with regulation from common signaling pathways such as TGFβ-smad3 and Wnt/β-catenin [40,41,42]. Changes in regulation of these pathways in addition to others has also been implicated in the pathogenesis and drug resistance of MBC [43,44]. The various subtypes of MBC express EMT transcription factors at varying levels, though regulatory change in EMT is seen to some degree in all MBC [45,46]. EMT may lead to overexpression of programmed death ligand (PD-L1), which allows cancer cells to evade the immune response [47]. This overexpression of PD-L1 has been reported in MBC and stands out as a therapeutical target [48]. One clinical trial found that treatment with Pembrolizumab, a PD1 inhibitor, showed modest response with no grade 4 adverse effects for patients with metastatic TNBC expressing PD-L1 [49]. Ongoing studies are investigating the efficacy of PD-L1 and PD-1 inhibitors in TNBC and show optimistic preliminary findings, though only one clinical trial including MBC is currently active (Table 4, NCT02834013). One case report found that combined therapy with nab-paclitaxel and Pembrolizumab incited a remarkable recovery in a triple-negative spindle-cell MBC patient with previously treated metastatic disease and overexpression of PDL-1, indicating that specific combinations of chemotherapy and immunotherapy may be more efficacious based on molecular and histological features of disease [50]. A current clinical trial is evaluating this combination in HER-2 negative metastatic breast cancer patients (Table 4, NCT02752685 [51]). Generalization of results in TNBC to MBC is questionable considering evidence distinguishing their pathogenesis and prognosis; however, the small incidence of MBC makes research a challenge. Understanding how specific molecularly aberrant tumors respond to targeted therapies in TNBC may still allude to efficacy in MBC with similar molecular findings. Information on specific genetic and molecular testing for each patient may provide physicians more insight into the efficacy of various treatment options in MBC. 

## 5. Limitations

The results of this study are limited by the completeness and accuracy of data in the SEER database, stemming from variances in provider documentation and the availability of patient data. Specific data regarding MBC subtype, type of chemotherapy used, sequencing of therapy, surgical intervention type, and margin status were not available, thus limiting the quality of the analysis. The SEER database only covers 48% of the US population, possibly missing important data from underrepresented geographic locations. Additionally, the results of this study may have limited generalizability to countries other than the US. 

## 6. Conclusions

Our study confirms the aggressive and treatment-resistant nature of MBC while indicating that high-grade tumors, distant metastasis, large tumor size, and advanced age were associated with poor prognosis. The Black race is associated with worse outcomes. Surgery with adjuvant chemotherapy and radiation therapy is the most effective means of treatment, though new advances in a personalized approach to the treatment of MBC may stand to increase patient survival. We believe clinicians can use the findings of this study to utilize genomic profiling and molecular testing to determine the best course of treatment and in turn contribute to the growing field of knowledge surrounding this rare but aggressive breast cancer. 

## Figures and Tables

**Table 1:** Demographic profiles and tumor characteristics of 5315 patients with metaplastic carcinoma of the breast from the Surveillance, Epidemiology, and End Results (SEER) database, 2000–2018.

Variable (n = 5315)			Frequency (%)
Age	18–29		46 (0.9%)
	30–39		256 (4.8%)
	40–49		690 (13.0%)
	50–59		1152 (21.7%)
	60–69		1243 (23.4%)
	70–79		1088 (20.5%)
	≥80		840 (15.8%)
Race	Unknown		25 (0.5%)
	White		4106 (77.3%)
	Black		833 (15.7%)
	Asian or Pacific Islander		324 (6.1%)
	American Indian or Alaska Native		27 (0.5%)
**Grade (n = 5315)**		**Frequency (%)**	
Unknown		1122 (21.1%)	
Known		4193 (78.9%)	
Grade where known (n = 4193)			
Well differentiated—Grade I		229 (5.5%)	
Moderately differentiated—Grade II		637 (15.2%)	
Poorly differentiated—Grade III		3122 (74.4%)	
Undifferentiated/Anaplastic—Grade IV		205 (4.9%)	
**Receptor status (n = 5315)**		**Frequency (%)**	
Triple negative		1978 (37.2%)	
HR+/HER2−		696 (13.1%)	
HR−/HER2+		107 (2%)	
HR+/HER2+		60 (1.1%)	
Borderline/Unknown		2474 (46.5%)	
**Variable (n = 5315)**			**Frequency (%)**
Stage	Unknown		435 (8.2%)
	Known		4880 (91.8%)
	Stage where known (n = 4880)		
	Localized		3286 (67.3%)
	Regional		1287 (26.3%)
	Distant		307 (6.3%)
Size	Unknown		1856 (34.9%)
	Known		3459 (65.0%)
	Size where known (n = 3459)		
	<20 mm		768 (22.2%)
	20–50 mm		1749 (50.6%)
	>50 mm		942 (27.2%)
Laterality	Left—origin of primary		2708 (51.0%)
	Right—origin of primary		2597 (48.9%)
	Bilateral—single primary		4 (0.1%)
	Only one side—side unspecified		1 (0.09%)
	Paired site—but no information concerning laterality		5 (0.1%)

**Table 2:** Selected variables by race data of 5315 patients with metaplastic carcinoma of the breast from the Surveillance, Epidemiology, and End Results (SEER) database, 2000–2018.

Variables	Race	
	White (n = 4106)	Black (n = 833)
Grade III	2359 (73.0%)	542 (80.5%)
Distant metastases	220 (5.8%)	72 (9.4%)
Tumor size > 50 mm	689 (25.8%)	193 (34.8%)
Positive lymph nodes	398 (21.1%)	102 (25.6%)
Bone metastasis	41 (1.8%)	15 (3.1%)
Brain metastasis	14 (0.6%)	2 (0.4%)
Liver metastasis	21 (0.9%)	8 (1.6%)
Lung metastasis	74 (3.2%)	25 (5.1%)
Triple negative	1531 (37.3%)	315 (37.8%)
HR+/HER2-	512 (12.5%)	108 (13%)
HR-/HER2+	76 (1.9%)	22 (2.6%)
HR+/HER2+	41 (1%)	12 (1.4%)
Borderline/Unknown	1946 (47.4%)	376 (45.1%)

**Table 3:** Multivariate analysis of independent factors influencing mortality of 5315 patients with metaplastic carcinoma of the breast from the Surveillance, Epidemiology, and End Results (SEER) database, 2000–2018.

Variables		Multivariate Analysis; Hazard Ratio (*p*-Value)
Age	>60	1.958 (0.001)
Grade	Undifferentiated/Anaplastic—Grade IV	3.692 (0.002)
Stage	Distant	2.613 (0.012)
Tumor size	>50 mm	3.275 (0.001)
Brain metastasis	Yes	29.266 (0.001)

**Table 4:** Ongoing treatment trials for metaplastic breast carcinoma (Source: Clinicaltrials.gov, accessed on 10 May 2023).

Trial Number	Study Title	Study Type	Intervention	Primary Outcome	Status
NCT02834013 [52]	DART: Dual Anti-CTLA-4 and Anti-PD-1 Blockade in Rare Tumors	Phase II, multicenter, Open-label	Arm 1: Nivolumab IV on days 1, 15 and 29 + Ipilimumab IV on day 1 for up to 17 42-day cyclesArm 2: Ipilimumab IV on days 1, 15, 29 for up to 17 42-day cycles	Overall response rate	Active

## Data Availability

The data that support these findings are housed with the Surveillance, Epidemiology and End Results Program (SEER) 18 registry dataset from 2000–2018.

## Supplementary Materials

The following supporting information can be downloaded at: https://www.mdpi.com/article/10.3390/cancers15112954/s1, Table S1: Regional lymph node status and distant metastasis at the time of diagnosis of 5315 patients 6 with metaplastic carcinoma of the breast from the Surveillance, Epidemiology, and End Results 7 (SEER) database, 2000–2018; Table S2: Treatment characteristics of 5315 patients with metaplastic carcinoma of the breast from 9 the Surveillance, Epidemiology, and End Results (SEER) database, 2000–2018; Table S3: Survival data of 5315 patients with Metaplastic carcinoma of the breast from the Surveil-11 lance, Epidemiology, and End Results (SEER) database, 2000–2018; Table S4: Survival by race data of 5315 patients with metaplastic carcinoma of the breast from the 13 Surveillance, Epidemiology, and End Results (SEER) database, 2000–2018.
