# Supplementary material for: Metaplastic Breast Carcinoma in U.S. Population: Racial Disparities, Survival Benefit of Adjuvant Chemoradiation and Future Personalized Treatment with Genomic Landscape
Source: Cancers (Basel). 2023 May 28;15(11):2954. doi: 10.3390/cancers15112954 (PMC10251813; doi:10.3390/cancers15112954)
Supplement: Supplementary file 1 [file cancers-15-02954-s001.zip › cancers-2304323-supplementary.pdf]

Supplementary Materials

Metaplastic Breast Carcinoma in U.S. Population: Racial Disparities, Survival Benefit of Adjuvant Chemoradiation and Future Personalized Treatment with Genomic Landscape

Table S1. Regional lymph node status and distant metastasis at the time of diagnosis of 5315 patients with metaplastic carcinoma of the breast from the Surveillance, Epidemiology, and End Results (SEER) database, 2000 - 2018.

**Nodal Status (n = 5315)**	**Frequency (%)**
Unknown	2823 (53.1%)
Known	2492 (46.9%)
When nodal status was known (n = 2492)	
Positive lymph nodes	551 (22.1%)
Negative lymph nodes	1941 (77.9%)
**SEER Metastasis (n = 5315)**	**Frequency (%)**
Unknown	2275 (42.7%)
Known	3040 (57.2%)
Where metastasis was known (n = 3040)	
No metastasis	2890 (95.1%)
Bone metastasis only	27 (0.9%)
Brain metastasis only	8 (0.3%)
Liver metastasis only	8 (0.3%)
Lung metastasis only	62 (2.0%)
Bone + Brain metastases	1 (0.03%)
Bone + Liver metastases	4 (0.1%)
Bone + Lung metastases	19 (0.6%)
Brain + Lung metastases	4 (0.1%)
Liver + Lung metastases	8 (0.3%)
Bone + Brain + Lung metastases	1 (0.03)
Bone + Liver + Lung metastases	5 (0.1%)
Brain + Liver + Lung metastases	1 (0.03%)
Combined metastases to bone, brain, liver, and lungs	2 (0.06%)

Overall Metastasis by Location

**Location**	**Status known (%)**		**Frequency (%)**
Bone	Unknown	2271 (42.7%)	59 (1.94%)
	Known	3044 (57.3%)	
Brain	Unknown	2268 (42.7%)	17 (0.56%)
	Known	3047 (57.3%)	
Liver	Unknown	2268 (42.7%)	30 (0.98%)
	Known	3047 (57.3%)	
Lung	Unknown	2270 (42.7%)	104 (3.42%)
	Known	3045 (57.3%)	

Table S2. Treatment characteristics of 5315 patients with metaplastic carcinoma of the breast from the Surveillance, Epidemiology, and End Results (SEER) database, 2000 - 2018.

**Treatment (n = 5315)**	**Frequency (%)**
Unknown	24 (0.5%)
Known	5291 (99.5%)
Where treatment status were known (n = 5291)	
No treatment	199 (3.8%)
Surgery only	1509 (28.5%)
Chemotherapy only	126 (2.4%)
Radiation only	6 (0.1%)
Surgery + Chemotherapy	1296 (24.5%)
Surgery + Radiation	574 (10.9%)
Chemotherapy + Radiation	11 (0.2%)

Combination therapy (Surgery + Chemotherapy + Radiation)	1568 (29.6%)

Table S3. Survival data of 5315 patients with Metaplastic carcinoma of the breast from the Surveil-lance, Epidemiology, and End Results (SEER) database, 2000 - 2018.

Survival	Overall Survival% (C.I. 95%)	Cause specific survival% (C.I. 95%)	Chemotherapy% (C.I. 95%)	Surgery% (C.I. 95%)	Radiation% (C.I. 95%)	Surgery + Chemotherapy (C.I. 95%)	Surgery + Radiation % (C.I. 95%)	Combined Chemotherapy, surgery, and Radiation (C.I. 95%)
1 year	88.4% (87.3-89.3)	90.1% (89.2-91.0)	92.2% (91.1-93.2)	92.7% (91.8-93.5)	96.0% (95.0-96.8)	93.9% (92.8-94.8)	96.1% (95.1-96.9)	96.8% (95.7-97.7)
2 years	77.9% (76.6-79.2)	81.4% (80.2-82.6)	82.7% (81.1-84.2)	84.1% (82.8-85.2)	89.1% (87.5-90.6)	84.5% (82.9-86.0)	89.4% (97.8-90.8)	89.2% (87.3-90.8)
3 years	71.4% (69.9-72.8)	76.2% (74.8-77.5)	77.6% (75.8-79.3)	78.8% (77.4-80.1)	83.6% (81.7-85.4)	79.4% (77.6-81.1)	83.9% (81.9-85.6)	83.3% (80.9-85.3)
4 years	67.1% (65.5-68.6)	73.1% (71.6-74.5)	74.3% (72.4-76.1)	75.7% (74.2-77.1)	80.5% (78.4-82.5)	76.3% (74.3-78.1)	80.8% (78.7-82.7)	79.8% (77.3-82.1)
5 years	63.6% (62.0-65.1)	71.1% (69.5-72.6)	72.6% (70.6-74.5)	73.6% (72.0-75.1)	78.7% (76.5-80.7)	74.5% (72.5-76.4)	79.0% (76.7-81.0)	78.1%(75.4-80.4)

Table S4. Survival by race data of 5315 patients with metaplastic carcinoma of the breast from the Surveillance, Epidemiology, and End Results (SEER) database, 2000 - 2018.

Survival	White% (C.I. 95%)	Black% (C.I. 95%)	Asian or Pacific Islander% (C.I. 95%)	American Indian or Alaska Native% (C.I. 95%)
1 year	90.3% (89.2-91.3)	88.8% (86.1-91.0)	91.6% (87.3-94.4)	85.0%% (59.7-91.5)
2 years	82.3% (80.9-83.7)	76.4% (72.8-79.6)	83.9% (78.4-88.1)	69.1% (43.6-84.8)
3 years	77.6% (76.0-79.1)	69.2% (65.2-72.8)	77.1% (70.8-82.2)	69.1% (43.6-84.8)
4 years	74.3% (72.6-75.9)	65.9% (61.8-69.7)	76.5% (70.2-81.7)	69.1% (43.6-84.8)
5 years	72.4% (70.7-74.1)	63.2% (58.9-67.1)	75.2% (68.7-80.6)	63.3% (37.9-80.6)
